# Sinunasal ultrastructure of the transplant hematopoietic stem cell and chronic graft-versus-host disease with rhinosinusitis

**DOI:** 10.1186/2045-7022-3-S2-O5

**Published:** 2013-07-16

**Authors:** Erica Ortiz, Afonso Celso Vigorito, Luciana R Meirelles, Eulalia Sakano, Ester Maria Daniele Nicola

**Affiliations:** 1 University of Campinas, Sao Paulo, Brazil, Otolaryngology Head-Neck, Campinas, Brazil; 2 University of Campinas, Sao Paulo, Brazil, Hematology, Campinas, Brazil; 3 University of Campinas, Sao Paulo, Brazil, Pathology, Campinas, Brazil

## Background

It is believed that immunosuppression is the sole cause for the occurrence of rhinosinusitis in hematopoietic stem cell transplant (HSCT). There is a high incidence of sinusitis in recipient's patients, especially those with Chronic Graft Versus Host disease (GVHD). Histopathological abnormalities were described in recipient's sinus mucosa comparing to the immunocompetents patients. There are also mucosal abnormalities related to the cytotoxicity in the transplanted patients with chronic GVHD, but no difference in ultrastructure between HSCT patients with and without GVHD, except increased goblet cells in patients without GVHD. The relation between the sinunasal mucosa abnormalities of patients with and without GVHD and rhinosinusitis is not well established yet.

## Objective

To verify the ultrastructure of the sinunasal mucosa of HSCT with and without GVHD with rhinosinusitis in order to understand the cause of high sinusitis incidence in recipients with and without GVHD.

## Method

A preliminary prospective study with statistical analysis of data obtained from the evaluation of the mucosa of the uncinate process by transmission electron microscopy of those recipients with (10) and without GVHD (9) with rhinosinusitis.

## Results

93% of transplanted patients with GVHD and 62% of those without GVHD had 2 or more rhinosinusitis. Only the presence of microvilli was significantly increased in patients without GVHD. There was no significant difference in the cilia number, cilia ultrastructure, squamous metaplasia, goblet cells or citoplasmatic vacuolization between those groups.

## Conclusion

The recurrence of rhinosinusitis seems to be higher in chronic GVHD patients, however no abnormalities were found in the ultrastructure of their sinunasal mucosa, except increased microvilli in those without GVHD. This is a preliminary study and an increased sample might modify statistical analysis, as well as the comparison of recipients without rhinosinusitis.

